# The Effects of the Activation of Money and Credit Card vs. that of Activation of Spirituality – Which One Prompts Pro-Social Behaviours?

**DOI:** 10.1007/s12144-014-9299-1

**Published:** 2014-12-24

**Authors:** Jakub Wierzbicki, Anna Maria Zawadzka

**Affiliations:** Psychology Institute of Psychology, University of Gdansk, ul. Bażyńskiego 4, 80-592 Gdańsk, Poland

**Keywords:** Money, Priming, Spirituality, Pro-social behaviour

## Abstract

Pro-social behaviours may be prompted or inhibited depending on the situation. Numerous experiments show that, when exposed to the idea of money, people are less willing to help, devote their time or share their resources with others (Vohs et al. Science, 314, 1154–1156, 2006, Current Directions in Psychological Science, 17(3), 208–212, 2008). Conversely, when exposed to the idea of spirituality, they often cheat less and are more willing to help others (Mazar and Ariely Journal of Marketing Research, 45, 633–644, 2008; Randolph-Seng and Nielsen The International Journal for the Psychology of Religion, 17(4), 303–315, 2007). The aim of this article is to present the results of two experiments in which we activated thoughts about money, i.e. both cash and credit cards, and thoughts about spirituality in order to find out in what way these two kinds of activation may influence pro-social behaviours. In experiment 1, participants, when reminded of money, offered lower donations to others whereas those reminded of spirituality offered higher donations. In experiment 2, those participants reminded of money offered to devote less time to help others whereas those reminded of spirituality offered to devote more time to help others.

## Introduction

There are good reasons why we may think that people behave differently towards others depending on whether they think about money or about spirituality. These interactions may be types of social relations in which people are involved, i.e. whether it is an economic exchange (based on the value of money) or a social exchange (based on mutual exchange of favours) (Fiske 1992). When people use money in social relations, their aim is to maximise profit, whereas altruism is the core of relations based on social exchange (Batson 1998; Cialdini 1997). Empirical continuation of the views presented above can be found in research on the universal structure of human values and goals. The results indicate that self-enhancement values (including wealth) are contradictory to self-transcendence values (including spirituality); in short, striving for wealth is contradictory to striving for spirituality (Grouzet et al. 2005).

Research based on priming participants for this proves that what people perceive (e.g. stimuli that people are exposed to) may influence their preferences and behaviour, even though they are not aware of this (cf. Bargh and Chartrand 2000). In view of this, it is interesting to examine how the effect of the activation of thoughts about money (i.e. priming with money) differs from the effect of the activation of thoughts about spiritually-related concepts (i.e. priming with spiritually-related concepts) on selected examples of pro-social behaviours in a specific situation.

The aim of this paper is to present the results of our study into the influence of the activation of thoughts about money and the activation of thoughts about spiritually-related concepts on pro-social behaviours. Pro-social behaviour is understood here as an activity done for the benefit of others (cf. Batson 1998).

Existing research demonstrates that the activation of thoughts about money inhibits pro-social behaviours (Gąsiorowska and Hełka 2012; Gąsiorowska et al. 2012; Vohs et al. 2006, 2008), and the activation of thoughts about spiritually-related concepts encourages pro-social behaviours as compared with a neutral situation (Mazar and Ariely 2008; Pichon et al. 2007; Randolph-Seng and Nielsen 2007; Shariff and Norenzayah 2007). Studies into the use of credit cards carried out so far indicate that when people use credit cards, as compared to cash, they want to buy more goods (Chatterejee and Rose 2012; Thomas et al. 2011).

The novelty of this study is that it analyses within a single study how pro-social behaviours are affected by both the activation of thoughts about money and the activation of thoughts about spiritually-related concepts. What’s more it is the first research to compare the effect of the activation of thoughts about abstract money, i.e. credit cards, with the effect of the activation of thoughts about real money, i.e. notes or coins, on pro-social behaviours.

Obtainable research shows that attaching importance to moral traits within the self-schema prompts people to act to benefit others (cf. Brewer and Gardner 1996). Therefore, we also looked at the relationship between the importance of moral traits within the self-schema and pro-social behaviours in the three contexts: the activation of thoughts about cash, credit cards and spiritually-related concepts.

## Theoretical Background

### Money, Social Relations and Pro-Social Behaviours – Theory and Research Review

The theory of social relations (Fiske 1992) includes four cognitive models that describe motivation, planning, production, comprehension, coordination and an evaluation of human social life. These models are: communal sharing, where people are interdependent, use common resources and expect mutual attention and care; authority ranking, where social relations are based on a linear hierarchy; equality matching, where people keep track of inequalities among participants in order to restore balance; and market pricing, where people make exchanges conforming with rational cost-benefit analysis. According to the theory, money is the archetype of the last model. It has an instrumental value in social relations. This value is based on entering into social relations following a profit and loss estimation. The main motivator for community actions is the motivation to achieve. However, in order to reach goals and feel motivated one needs a sense of self-efficacy (Bandura 1977). The essence of self-efficacy consists of achievements in one’s actions, i.e. successes and failures, and testing one’s capabilities as well as the efficiency of one’s behaviours in new and difficult situations. Based on the theory of social relations, researchers assumed that money is linked to self-efficacy in social relations (Vohs et al. 2006). The feeling of self-efficacy coexisting with the use of a profit and loss estimation in social relations makes people think they need resources in order to undertake and perform tasks and so feel reluctant to let others participate in those actions (Vohs et al. 2008). Nowadays, money is associated with numerous types of positive situations and objects, e.g. food, drinks, being accepted by others or social status, which have a high gratification value (Baryła 2013). Images of money and symbols of currency can activate the pleasure-seeking centre and people recognize them automatically (cf. Knutson et al. 2003; Tallon-Baudry et al. 2011). Furthermore, the brain mobilizes the body to get the reward represented by a certain amount of money and adjusts the level of mobilization to the value of the reward (Baryła 2013; Pessiglione et al. 2007). This mobilization preceding instrumental behaviours leads to a temporary accumulation of motivational potential and, as a result, to its consumption by other decisions or behaviours. The subject is motivated to perform these when signals releasing motivational mobilization occur (Baryła 2013). Energy transfer lays the base for the model of motivational consequences of the activation of thoughts about money.

In a series of experiments on the effect of money-priming on pro-social behaviour, Vohs and her collaborators found that money-primed subjects were less likely to offer help, less likely to ask for help, and more likely to choose an individual activity over a group activity (Vohs et al. 2006, 2008). Interestingly, two aspects of helping behaviour based on two different resources, i.e. time and money, were examined. In one of the experiments the participants completed a descrambling task, i.e. created sensible phrases out of five jumbled words. In the money condition, 15 phrases were money-related and 15 phrases were neutral. In the control condition, all 30 phrases were neutral. Next, participants completed some inconsequential questionnaires after which followed the significant part of the experiment. This tested how likely they were to help, that is to explain the directions for the task to another participant. The measure of help was the time spent explaining. The experiment showed that participants primed with money spent half as much time helping as did the participants in the control condition (Vohs et al. 2006). Similar results were obtained in another experiment designed to test how likely the participants were to help with regard to money. The same money-priming method was used, but the participants were given two dollars apiece in quarters for their work. When they were about to leave, the experimenter told them they were welcome to make donations to a university student fund. It was found that participants primed with money offered significantly less money (39 % of the two dollars) than those who were not primed with money (67 % of the two dollars) (Vohs et al. 2006, 2008).

It might be supposed that money-priming makes people less social towards strangers, but not family or friends. In yet another experiment (Vohs et al. 2008), participants were randomly divided into three groups, seated at desks and asked to complete meaningless questionnaires. In the money condition, the desks faced posters with photos of various denominations of currency, whereas in both control conditions the desks faced posters with seascapes or flower gardens. Next, they had to choose between two leisure activities (one to be enjoyed by one person only and the other by two or more) using a nine-item questionnaire. Participants primed with money chose more individually-focused options than participants in the two control conditions.

Other experiments (Gąsiorowska et al. 2012) were conducted on children aged 5–8 who were primed with money through play with specimens of money. The findings showed that children exposed to money were more selfish while playing economic games, less likely to display pro-social behaviours and less likely to help the experimenter than children in the control group. Interestingly, although the children did not understand the economic or instrumental meaning of money but only its symbolic meaning, the mere concept of money affected their pro-social behaviours.

The concept of money also affects adults’ behaviours in economic games such as the dictator game, for example. In one of the experiments, participants counted various objects shown on the computer screen. The objects included coins in the money condition and they included round candies resembling coins in the control condition. Next, they played two rounds of the dictator game (once as the proposer and once as the responder) and then they completed debriefing questionnaires. Participants primed with money transferred less money in the game than participants in the control group. What is more, participants primed with money playing the role of the proposer expressed stronger negative emotions and were less satisfied with their decisions than those in the control condition who had made similar transfers (Gąsiorowska and Hełka 2012).

Previous research compared how implicit activation of the concept of money and implicit activation of the concept of time affect pro-social behaviours. The findings show that individuals primed with money, as opposed to those primed with time, cheat more when given the opportunity to do so (Gino and Molinger 2014). Researchers explain that this may be due to the fact that time-priming makes people reflect on who they are and cheat less as a result. Furthermore, existing research also reveals that the presence of abundant wealth in the environment in which people function may increase the temptation to cheat (Gino and Pierce 2009). Laboratory studies (anagram tasks) proved that exposure to huge amounts of money, as opposed to small amounts, may lead to engagement in dishonest behaviour for financial gain, e.g. overstating their performance.

Research referring to Friske’s relational theory (1992) revealed that abstract money introduced into social relations may have a similar effect to that of real money. Research was carried out in order to find out how willing people are to help when they receive three different forms of payment: cash, candies or ‘monetised’ candies (candies whose cost is known – abstract money). The results indicated that people were always willing to help when they received candies for their work. However, when the people were rewarded with cash or ‘monetised’ candies their willingness to help decreased and depended on the amount they received (Heyman and Ariely 2004). For that reason, it may be concluded that abstract money (‘monetised candies’) affects pro-social behaviours in the same way as real money does.

Therefore, we assumed in our study that both real money (cash) and abstract money (credit cards) will affect a willingness to help, i.e. decrease willingness to help.

### Spirituality, Social Relations and Pro-Social Behaviours - Theory and Research Review

Fiske (1992) contrasts the relational model based on economic exchange (money) with relational models based on social exchange. Social exchange models assume that the main motive for pro-social behaviours is altruism (Batson 1998; Cialdini 1997), which is the primary principle of social relations preached by the main religious systems. Available research (Pichon et al. 2007) demonstrates that positive spiritual priming increases pro-sociality. Namely, participants were primed with spiritually-related words, both positive (e.g. heaven, miracle, salvation) and neutral ones (e.g. incense, altar, rosary). Next, the researchers assessed the accessibility of pro-socially related items (e.g. support, help) in a lexical decision task. It was found that participants exposed to positive spiritual primes identified pro-social items faster.

In recent years, there has been a growing interest in studies demonstrating that the activation of spiritually-related concepts affects pro-social attitudes and behaviour, such as honesty, a willingness to help or a willingness to share. The research suggests that people tend to be more pro-social when they think about spiritually-related concepts than when they do not. In one study participants were primed with a scrambled sentence task: spiritually-related, sport or neutral theme (Randolph-Seng and Nielsen 2007). Next, they had to complete a circle test; they were induced to cheat as they could win extra credit while performance expectations were unrealistic. Moreover, the level of religiosity of the participants was also measured. The results showed that activation of spiritually-related concepts led to an increase in honesty. However, no link was found between the level of religiosity and honesty of the participants. In other words, it was not religious conviction that affected the level of honesty but rather exposure to spiritually-related concepts.

Researchers also examined how individuals with high regard for moral traits within the self-schema behave when they are primed with spiritually-related concepts. The findings indicate that when situational factors make moral traits more accessible within the self-schema, both the intention to behave pro-socially and pro-social behaviours themselves increase (Aguino et al. 2009). Participants in one group had to list the Ten Commandments, while in the other they had to list the largest cities in the U.S. Next, the participants were asked to imagine they were brand managers of a product and declare whether they were willing to donate a few cents to a special fund for cancer prevention each time the product was purchased. The results indicated that priming of spiritually-related concepts increases accessibility to the moral self-schema for individuals with a low regard for moral traits within the self-schema; This increases a willingness to help as compared with participants with high regard for moral traits within the self-schema.

Another study tested how spiritually-related concepts influence cheating (Mazar and Ariely 2008). Participants had to recall either the Ten Commandments or the titles of ten books they had read at secondary school. Next, they had to solve matrix tasks within a time limit. Subsequently, they were also told that two randomly selected participants would earn $10 for each correctly solved matrix. At the end of the matrix task, participants in these experimental conditions were provided with an opportunity to cheat since they only had to indicate the total number of correctly solved matrices without further verification of their answers. The results indicated that when given the opportunity to cheat, participants did so after a book recall task but did not cheat after the Ten Commandments recall task.

In a series of studies researchers examined whether spiritual priming may facilitate cooperation (Shariff and Norenzayan 2007). In the first study, one of two randomly-assigned groups was primed with spiritually-related concepts using scrambled sentences while the other was not. Afterwards, the participants played one round of the anonymous version of the dictator game. They were given 10 one-dollar coins each and their role was to keep or give to an anonymous player as many as they choose to. It was found that those in spiritual-prime condition left more money to the anonymous player than those in the control condition. Furthermore, this effect was present for both believers and atheists. The second study was aimed at replicating and extending the findings of the first study. Participants were assigned to three conditions: neutral prime, secular prime and spiritual prime and followed a procedure similar to that of study one. Once more, the results showed that participants in spiritual-prime condition offered more money to the anonymous player as compared to the other two conditions. Additionally, the effect was strongest for those who declared themselves believers. All in all, although spiritual priming increased generosity in economic games, the level of religiosity did not correlate with generosity.

Other research is available that looked into the question of how a spiritual venue as compared to a secular venue may affect willingness to cooperate (Xygalatas 2012). Participants were randomly assigned to two groups: one was sent into a venue for religious worship and the other into a restaurant. They played a bargaining game (a common-pool resources game) in which some level of cooperation was required. They had to decide how much money to withdraw from a shared account, but their return depended on the request of the other player. It was found that participants playing inside the spiritual venue were more cooperative than those playing in the secular one. The level of religiosity did not parallel the level of willingness to cooperate.

In view of the above conclusions concerning the effect of either the activation of thoughts about money or the activation of thoughts about spiritually-related concepts on pro-social behaviour, we assumed that money (cash and credit card) priming would decrease while spiritual priming would increase pro-social behaviour, i.e. willingness to help.

### Moral Traits Within the Self-Schema vs. Pro-Social Behaviour

Researchers established that the two universal dimensions that people use to perceive themselves and others are morality (socially good vs. bad) and efficiency (intellectually good vs. bad) (Rosenberg and Sedlak 1972; Wojciszke 2005). These dimensions are of great importance in building up self worth. Research indicates that being moral is both a valuable and desired trait for others (Wojciszke et al. 1998). Moral categories are more important in the interest of others, Thus possessing moral traits is beneficial for others. The concepts of morality and efficiency as dimensions of the self-schema are semantically close to dependent and independent self-construals. According to available research, individuals with a dominant dependent self-construal focus more on relationships with others while individuals with a dominant independent self-construal focus more on their own achievements and success (Brewer and Gardner 1996; Marcus and Kitayama 1991). In other words, those with a dependent self-construal (semantically referring to the central role of moral traits within the self-schema) define themselves in terms of relationships with others. Therefore, they prefer close contacts with others and have a pro-social orientation

Researchers’ findings indicate that the importance of moral traits in the self-schema may be linked to pro-social behaviours (Aguino and Reed 2002; Hart and Fegley 1995; Johnston and Krettenauer 2011). Correlational research on adolescents revealed that when moral values are important within the self-schema the willingness to undertake pro-social behaviour increases (Johnston and Krettenauer 2011). Other research showed that individuals personally involved in voluntary work described themselves using a greater number of moral traits than those who did not do voluntary work (Hart and Fegley 1995; Hart et al. 1995). Another study examined how the weight of selected moral traits (e.g. helpful, sympathetic, just, friendly) is related to intentions to participate in voluntary work. It was found that individuals who set a higher importance on moral traits in the self-schema were more willing to participate in voluntary work as compared to those for whom moral traits were less important (Aguino and Reed 2002).

Therefore, we assumed in our analysis that individuals who place higher importance on moral traits in the self-schema will be more willing to help (share their time and money) than those within whose self-schema moral traits are less important.

### Hypotheses

In light of the research presented above demonstrating that pro-social behaviours decrease when thoughts about money are activated and increase when thoughts about spiritually-related concepts are activated, we formulated the first hypothesis:

1. Activation of thoughts about cash and credit cards inhibits pro-social behaviours (declared amounts of money and time devoted to others) while activation of spiritually-related concepts prompts pro-social behaviours as compared to the control condition.
  Consistent with the findings, indicating links between the importance of moral traits within the self-schema and pro-social behaviour, we framed the second hypothesis:
2. Individuals who place a higher importance on moral traits in the self-schema will display more pro-social behaviours (declare bigger amounts of money and more time devoted to others) than those for whom moral traits are less important.
  The hypotheses were tested in two experiments presented below.

## Experiment 1: Willingness to Help – the Amount of Donated Money

In the first study, we tested how the activation of thoughts about cash, credit cards and spiritually-related concepts influences the amount of donated money.

## Method

### Participants

One hundred nineteen participants (average age *M* = 28.8 (SD = 7.29), 81 females and 38 males) took part in the study. They were full-time and part-time students of the Adam Mickiewicz University in Poznan, who participated in the study to acquire course credits. The survey was carried out in groups of 10 to 15 students and took about 45 min. Cash-prime, spiritual prime and control conditions involved 30 participants each and credit-card prime condition involved 29 participants.

### Procedure and Materials

#### Neutral Independent Variable

In order to measure the importance of morality in the self-description a List of Traits was used (Wojciszke et al. 1993). This presents 23 traits, 8 of which refer to competence (e.g. clever, intelligent, creative), 7 refer to morality (e.g. understanding, loyal, honest) and 8 are neutral (e.g. cheerful, nice, good-looking). Participants indicated their answers on a scale from 1 to 11, where 1 = It does not describe me at all and 11 = It describes me perfectly. The level of the importance of moral traits in the self-description was demonstrated by the overall importance of these rates in the items in the List of Traits referring to morality. The internal consistency, Cronbach’s alpha, of these moral traits was *α* = 0.87.

#### Manipulated Independent Variable

Conceptual priming technique was applied. This activates cognitive representations in a specific context in order to influence the perception of a different context (cf. Bargh and Chartrand 2000). The activation of thoughts about cash, credit cards and spiritually-related concepts was performed with materials used previously by other researchers (Vohs et al. 2006, 2008; Gąsiorowska et al. 2012; Chatterjee and Rose 2012). Participants were randomly assigned to four groups. Group one arranged various credit cards (22) according to their subjective perceived value. Group two sorted banknotes (43) according to their currencies. Group three matched pictures of well-known saints (10, male and female) with descriptions of their spiritual lives. Group four sorted coloured paper cards (87) by colour (7).

#### Dependent Variable

In the last part of the experiment all students were informed that they would receive some money (PLN 30) for their participation in the study, after which they were asked to make a donation to an existing student association at their university to finance a study tour. Finally, they were asked to declare in writing how much money they were willing to contribute.

## Results

### Condition vs. Willingness to Donate

First, we ascertained how often participants in the experimental conditions declared a willingness to donate money to others. A chi-squared test indicated a difference between the groups showing a tendency towards importance *χ*^2^(2) = 4.16, *p* < 0.10; 11 out of 30 persons in the money condition, 11 out of 29 persons in the credit card condition and 18 out of 30 persons in the spiritually-related concepts condition. Participants from the first two groups declared a willingness to donate money less frequently than in the spiritually-related concepts condition. In the control condition 15 out of 30 persons declared a willingness to make a donation.

### Condition and Morality Importance Within the Self-Schema vs. Declared Amounts

Next, we verified how much money the participants were willing to donate. A regression analysis with categorical variables was performed. This model allows for an analysis of relationships between dependent variable and mixed independent variables, i.e. categorial and continuous. In order to adjust the variables entered into the regression model in order for them to meet its requirements, the nominal variable, i.e. the condition, was converted into three instrumental variables by binary coding (zero–one). The first variable, called *cash*, represented the group primed with real money. The second variable, called *card*, represented the group primed with credit cards. The third variable, called *spirituality*, represented the group primed with spiritually-related concepts. The control condition was the reference group. Independent variables included newly-created instrumental variables concerning the conditions of cash, credit cards and spiritual activation, the importance of morality within the self-schema and interactions between the conditions and the status of morality within the self-schema. The dependent variable was the declared donation amount. The tested model proved significant: F(7, 111) = 5.88, *p* < 0.001. The tested variables explained R^2^ = 0.27 variance of the dependent variable of the declared amount in the tested sample.

Regression analysis coefficients β indicated that the condition in which spiritually-related thoughts were activated differed significantly from the control condition (*spirituality* - *β* = 0.38, *t* = 3.57, *p* < 0.001), and that the more important morality was within the self-schema in this condition, the lower the donation amounts declared (*spirituality x morality within self-schema* - *β* = −0.54, *t* = −4.55, *p* < 0.001) (cf. Table 1). The results show that participants declared higher donation amounts when influenced by spiritually-related thoughts, but at the same time participants who placed a higher emphasis on morality within the self-schema declared lower amounts when influenced by spiritually-related thoughts.

**Table 1:** Regression analysis: conditions, morality importance within the self-schema, and interaction of conditions and morality, dependent variable – declared donation amount (experiment 1)

Variables	*Β*	*t*
Cash Cards Spirituality Morality within self-schema Cash × morality within self-schema Cards × morality within self-schema Spirituality × morality within self-schema	−0.04 −0.05 0.38 0.03 0.004 −0.003 −0.54	−0.38 −0.52 3.57*** 0.22 0.04 −0.03 −4.55***
	*R* = 0.52, R^2^ = 0.27 *F* = 5.88 (df = 7.111)	

*N* = 119; significance level - *p* < 0.001***

Hypothesis H1 was confirmed. Specifically, activation of spiritually-related concepts influenced the reaction of pro-social behaviours as compared to absence of activation (control condition). However, activation of thoughts about cash or credit cards had no effect on the declared donation amounts as compared to control condition. Hypothesis H2 was not confirmed. The importance of morality in the self-schema did not statistically (significantly) affect declared donation amounts.

## Experiment 2: Willingness to Help – the Amount of Time Devoted to Others

In order to verify the relationships assumed in study 1, we carried out another study. A different dependent variable relating to pro-social behaviour, i.e. time devoted to help others, was introduced. Thus, study 2 tested how the activation of thoughts about cash, credit cards and spiritually-related concepts influences people’s willingness to devote time to helping others and how the importance placed on morality within the self-schema is linked with the amount of time devoted to others.

## Method

### Participants

One hundred fifty eight participants (average age *M* = 24.34, SD = 4.39), 92 females and 66 males) took part in the study. They were full-time and part-time students of the Adam Mickiewicz University in Poznan, who participated in the study to acquire course credits. The survey was carried out in groups of 10 to 15 students and took about 45 min. Each situation: credit-card prime, spiritual-prime and control group involved 36–39 participants each. The cash-prime condition included 46 participants.

### Procedure and Materials

#### Non-Manipulated Independent Variable

In order to measure the importance of morality in self-description, the same List of Traits was used as in study 1 (Wojciszke et al. 1993). The level of the importance of moral traits in self-description was demonstrated by cumulative importance rates of all moral traits included in the List. The internal consistency, Cronbach’s alpha, of the importance of cumulative moral traits was *α* = 0.82. Next, to buffer the effect of the task, participants were asked to give 5 reasons for learning a foreign language at university.

#### Manipulated Independent Variable

Participants undertook a scrambled sentence quiz. They made 5-word sentences out of 6 words on sheets of paper specially prepared for each condition. In the cash prime condition participants had to make 15 sentences about cash (e.g. scrambled sentence - “wallet banknotes into balcony fit the”, correct sentence - “banknotes fit into the wallet”) on a sheet of paper on which were printed three images of Polish coins and banknotes. In credit card prime condition participants had to make 15 sentences about credit cards (e.g. scrambled sentence – “lost credit card desk I my”, correct sentence – “I lost my credit card”) on a sheet of paper with four images of credit cards. In spiritual prime condition participants had to make 15 sentences about spirituality, helpfulness and holiness (e.g. scrambled sentence – “went I church snow yesterday to”, correct sentence - “I went to church yesterday”) on a sheet of paper with three images of saints. In control condition participants had to make 15 neutral sentences (e.g. scrambled sentence – “outside is mountain today it cold”, correct sentence - “it is cold outside today”) on a sheet of paper with three images of nature on it.

#### Dependent Variable

After priming, participants were asked to volunteer to help an association “Child Without Ads” organize a talk for other students from the same university and indicate, on a monthly calendar, the weekend, time and number of hours which they wanted to devote to the cause. The measure of the dependent variable was the declaration of willingness to help (yes or no) and the amount of time that participants were willing to devote.

## Results

### Condition vs. Willingness to Devote Time to Others

First, we analysed how often participants in all conditions (i.e. cash, cards, spirituality and control groups) declared a willingness to help (devote their time) to organize the talk. The analysis demonstrated that there was a statistically significant difference in the number of persons declaring willingness to devote time to others in all four conditions: *χ*^2^(3) = 15.19, *p* < 0.01. Participants in spiritual prime declared a willingness to devote time to others most often (11, *N* = 37); participants in control group declared a willingness to devote time to others less often than in the previous condition (8, *N* = 26); participants in the two remaining conditions declared a willingness to devote time to others least often, cash prime (2, *N* = 46) and cards prime (2, *N* = 37). Therefore, the results confirm hypothesis H1 – there is more willing to devote time to others when the onus is on spiritually-related concepts. They are less willing to do so when they think about cash as compared to neutral thoughts.

To verify the hypotheses we carried out a linear regression analysis (enter method). The variable concerning the three conditions was converted into three new instrumental variables, as in study 1, and given the following names: cash (money prime), cards (credit cards prime) and spirituality (spiritually-related concepts prime). The control condition was the reference group. The tested model proved statistically significant: F(7, 150) = 4.006, *p* < 0.001. After entering the variables, the types of activated concepts (cash, credit cards, spiritually-related concepts), morality importance in the self-schema and interactions of those variables, the overall R^2^ relating to the declared amount of time devoted to helping others was 0.16. Regression coefficients analysis indicated that participants primed with credit cards differed significantly from non-primed participants in the amount of time declared to helping others (*β* = −0.23, *t* = −2.34, *p* < 0.05) (cf. Table 2). Specifically, they were willing to devote less time to help others. The store set on morality in the self-schema influenced the willingness to declare time to helping others – the higher the importance of morality the less willingness to declare time to helping others (*β* = −0.039, *t* = −2.05, *p* < 0.05). Coefficient β for conditions interactions was also statistically significant: activation of cash and credit cards x importance of morality in self-schema (cash x morality in self-schema *β* = 0.31, *t* = 2.62, *p* < 0.01; cash x morality in self-schema *β* = 0.22, *t* = 1.70, *p* < 0.09 – significance on tendency level). This means that participants who were primed with cash and credit cards and had a high sense of morality in the self-schema were willing to devote more time to helping others.

**Table 2:** Regression analysis: conditions, morality importance within the self-schema, and interaction of conditions and morality; the dependent variable – the declared amount of time devoted to others (experiment 2)

Variables	*β*	*t*
Cash Cards Spirituality Morality within self-schema Cash × Morality within self-schema Cards × Morality within self-schema Spirituality × Morality within self-schema	−0.14 −0.23 0.05 −0.39 0.31 0.22 −0.06	−1.38 −2.34* 0.49 −2.05* 2.62** 1.70 (*p* < 0.09) −0.48
	*R* = 0.40, R^2^ = 0.16 *F* = 4.01 (df = 7.150)	

*N* = 158; significance level - *p* < 0.05*, *p* < 0.01**

Hypothesis 1 was confirmed. Specifically, participants primed with credit cards were willing to devote less time to helping others than those in the control group. As for hypothesis 2, the results indicated a relationship contrary to the assumed hypothesis – a high regard for morality in the self-schema paralleled low pro-social behaviour.

## Discussion and Conclusions

The results of both studies demonstrate that the activation of thoughts about credit cards has a similar effect to that of the activation of thoughts about cash – it inhibits pro-social behaviours as opposed to the activation of thoughts on spiritually-related concepts, which prompts pro-social behaviour. In the presented studies, we examined types of pro-social behaviours that have not been analysed so far: a willingness to donate money and devote time to helping others. Available research has already looked at the question of whether it is the concept of money or spiritually-related concepts that influence a willingness to cooperate, helping others and honesty (cf. Gąsiorowska et al. 2012; Vohs et al. 2006, 2008; Randolph-Seng and Nielsen 2007; Shariff and Norenzayan 2007). In experiment 1, the activation of both cash and credit cards resulted in lower donations to others whereas the activation of spiritually-related concepts resulted in higher donations. In experiment 2, the effects were similar – the activation of both cash and credit cards resulted in a lesser amount of time declared in helping others whereas the activation of spiritually-related concepts resulted in a greater amount of time. The results, confirmed in two studies, have important social implications – abstract money (credit cards) inhibits the examined types of pro-social behaviour, similarly to the effect of real money (cash) on pro-social behaviour. The results of the studies presented here support previous research. Researchers established that when people are rewarded with real money (cash) or abstract money (candies resembling coins or ‘monetised’ candies) for pro-social behaviours, they form interpersonal relations following rules of economic exchange – they aim at benefit maximization (the willingness to help depends on the amount of money they receive for this) (Heyman and Ariely 2004).

The experiments we conducted did not confirm the assumptions concerning the relationship between the status given to morality in the self-schema and pro-social behaviour. Therefore, the results support previous findings. Existing research demonstrates that situational activation of spiritually-related concepts prompts pro-social behaviours whereas religiosity has little effect on pro-social behaviours or does not affect them at all (Randolph-Seng and Nielsen 2007; Shariff and Norenzayan 2007). The second experiment showed that the activation of the concept of money in the minds of people with high sense of morality in the self-schema prompted pro-social behaviour, as originally assumed. However, the activation of spiritually-related concepts in the minds of people with high regard for morality in the self-schema inhibited them from wanting to devote time to helping others. These results support the results of previous studies showing that spiritually-related concepts may influence pro-social behaviour of subjects with a low regard for morality in the self-schema but do not affect subjects with a high regard for morality in the self-schema (cf. Aquino et al. 2009). The results indicating that people with the latter in the self-schema were low in social behaviour when primed with spiritually-related concepts may be explained by “moral self-licensing” effect. It suggests that a high opinion of one’s own morality (past good deeds) can exempt individuals from engaging in pro-social behaviours (Merritt et al. 2010).

The results of both studies suggest that the activation of a specific concept (a specific semantic representation) influences behaviours in accordance with the linked representation (Bargh and Chartrand 2000). Thus, we may presume that thinking about money activates thinking about interpersonal relations as trade exchange, increases the sense of self-sufficiency and inhibits pro-social behaviours whereas thinking about spiritually-related concepts activates thinking about interpersonal relations such as social exchange, increases the sense of affiliation and prompts pro-social behaviours. The results of the studies presented here may be interpreted with regard to universal structures of human values and goals including stimuli that symbolise these values (Grouzet et al. 2005). In this way, activated stimuli in the domain of self-transcendence (spiritually-related concepts) and in the domain of self-enhancement (money) alone have a reverse effect on pro-social behaviours. The activation of the concept of money and credit cards may inhibit a willingness to involve mutual help whereas the activation of spiritually-related concepts prompts it.

In the studies presented here, participants declared an intention to help at a later time. Future research should examine how the activation of thoughts about money and thoughts about spiritually-related concepts affect pro-social behaviours performed immediately after priming. Another interesting question to answer would be whether there are universal spiritually-related concepts prompting pro-social behaviours regardless of culture and religion. It would also be interesting to discover whether the concept of credit card activates a sense of self-sufficiency, just as money does (cf. Vohs et al. 2006, 2008), or whether there is a different mechanism responsible for the influence of activation of credit cards on pro-social behaviours.
